# Annus Medicus

**Published:** 1905-12-30

**Authors:** 


### Hospital Clinics.

### ANNUS MEDICUS.

It cannot be said that any discoveries of the first rank have been made during the last year or that the progress of any great plague has been at last stayed. Indeed, the diseases for which specific remedies were promised only a few years back continue vigorously, and few of them show signs of disappearance. The bubonic plague itself has for the last three years destroyed a million of our Indian fellow subjects annually in spite of the exact bacteriology of the present time, and none of the sera or suggested remedies have any practical value in lessening its ravages. We are threatened again with outbreaks of cholera and epidemic meningitis. The latter, which destroyed 1,800 people in New York in the early spring, was also epidemic in Europe, and some eight years ago ravaged Massachusetts, and has been known for a century, but no reliable remedy has been found, though large doses of diphtheria antitoxin have given good results in a few cases. A warning has been issued by the Local Government Board that care should be taken lest outbreaks of it be overlooked, and especially those irregular cases which are often met with. The circular rightly insists on the importance in diagnosis of lumbar puncture and the detection in the fluid of the diplococcus intracellularis. Indeed, lumbar puncture and the study of the cells in spinal and similar fluids have obtained final acceptance means of distinguishing several important diseases, and as valuable aids to clinical medicine. The investigation of tropical diseases has been conducted with great energy; but here again progress, it must be confessed, is very slow. The ravages of sleeping sickness and the various trypanasome diseases have as yet been unchecked in British East Africa and elsewhere, though great advance has been made in our knowledge of the pathology of these parasitic forms of disease. Ankylostomiasis, which has recently begun to invade this country, has shown more successful results, and has been almost annihilated in parts of Germany, though the actual expenditure on the efforts has amounted to nearly a quarter of a million sterling.

The leprosy bacillus has once more disappointed our hopes. Not only was it announced that a suitable medium had been found for its growth, but a serum was obtained which it was hoped would check the ravages of the disease. The close resemblance of the bacillus to other acid-fast organisms seems to have led to error, and we are still unable to do more than isolate the sufferers.

*Food Questions.*—Not only has the work of Pawlow begun to bear fruit in a more rational theory of digestive metabolism, but the old estimates of a working diet have been widely questioned by Chittenden and the American school, and the proteid required for active work has been shown to be less than was believed to be necessary, though the final decision of the question has by no means been arrived at. The questions of physical deterioration and the low birth-rate have led to much work being done both on infant dietary and the improvement of the milk supply either by the action of local authorities or by private societies. The numbers of underfed children which were being driven into the schools have at last caused a minute from the Local Government Board directing Boards of Guardians to act in the matter.

As regards *Röntgen rays* the great hopes which were entertained of a remedy for cancer have yielded only a very limited degree of success, but the rays have certainly not fallen into disuse. Besides their efficacy in lupus some good results have been obtained in myelogenous leukemia and certain other diseases of that type, while their practical utility in ringworm is by no means a matter of little importance.

*The Prevention of Apoplexy.*—Great advances have been made in the study of high blood pressure and the need of its early recognition. The introduction of easy and reliable means of measuring the blood-pressure has enabled us to discover dangerous tensions where they were hitherto unsuspected. To Professor Allbutt and Oliver is due much of the interest which has been excited in this search for a means of preventing cerebral hæmorrhages.

The treatment of pulmonary tuberculosis has been prominently brought to our notice by the International Congress of Tuberculosis at Paris, but this meeting was only one of the many public gatherings dealing with the "white man's scourge." Not the least noteworthy was a deputation to the Metropolitan Asylums Board in April to suggest that an order of the Local Government Board should be obtained to make Section 5 of the Metropolitan Poor Act of 1891 applicable to pulmonary phthisis. Those who spoke in support of the proposal urged that pulmonary consumption would thereby in course of time be on the way to total extirpation, and Sir William Broadbent thought it probable that by sanatorium treatment a large proportion of the sufferers would be restored permanently to health. We doubt whether sanatorium treatment on a large scale for the treatment of poor consumptives would pay, but that, after all, must be only a secondary consideration. We have a duty to perform towards our poorer consumptives, and it may be that the cost of arresting the diseases may be greater than many realise, considering the length of time required for sanatorium treatment to restore even favourable cases to health and strength, but it seems certain that means will have to be adopted for dealing with large numbers of the phthisical poor on sanatorium lines. This, however, must be supplemented by measures for improving the hygienic conditions under which the poor live, and it is quite possible that the greatest benefit derived from the establishment of phthisical sanatoria for the poor may be the indirect result of educating that class in the value of fresh air and a life devoid of many of the avoidable baneful influences in the average daily life of the poor. Expectations were raised by the announcement that Professor von Behring had devised a remedy which he believed was efficacious in arresting and curing tuberculosis, but as yet the investigations have not been carried far enough to warrant more than the hope that he is proceeding on lines which will give to suffering humanity benefits that may equal the discovery of diphtheria antitoxic serum.

In laryngology the most striking event in the past year was the Garcia centenary, and the ceremony brought vividly before us the immense advances in the art of medicine that may ensue from a discovery which at the outset seemed to warrant little but an inspection of the vocal cords for the interest of singers. Manuel Garcia, a teacher of singing, sought means for inspecting the vocal cords and by a sudden inspiration conceived the idea of reflecting sunlight down the throat by means of a dental mirror with a long handle. His paper describing the methods and the results was fortunately brought under the notice of Turck, of Vienna, who had himself meanwhile devised a laryngoscope, and soon after the laryngoscopic observations were taken up by Czermak. It is needless nowadays to enlarge on the immense value and importance of these inventions, but it was most fitting that the attainment of his 100th year should be made the occasion of congratulations to Manuel Garcia, while no one who took part in the proceedings could have failed to be impressed with the remarkable and pleasing spectacle of a centenarian taking so active a share in the ceremonies.

Turning now to Surgery and its progress during the past year, perhaps the most striking advance has arisen in *Neurological Surgery*. The arrangement of the work at Johns Hopkins Hospital is such that the master minds can direct their junior colleagues into certain lines, and, by letting them have plenty of clinical material in the particular subject at which they are working, a great deal of much valuable original work has been added to the medical world. Thus, under Halsted, Harvey Cushing has been given lately most of the nervous work. He has published numerous cases of trephining for fractured base with symptoms of compression, and, apart from any definite localising sign, the relief of tension, the letting out of clot, and the avoidance to some degree of traumatic œdema, have led sometimes to surprisingly good results. He operates, when for decompressive purposes only, through the temporal fossa, removing the squamous position of the bone, and leaves the dura open after slitting it up in a stellate fashion, the muscle helping considerably afterwards to avoid fungus cerebri.

Many cases of stillborn children or those dying soon after birth having been found to have intracranial hæmorrhage, he has on several occasions exposed the brain and washed out subdural clot in cases which were obviously dying with a majority of satisfactory results. Even internal capsular hæmorrhage has been tackled, and more than one life saved. Washing out the base of the brain in meningitis has also been followed by recovery.

Perhaps the most ingenious of his new operations is that for chronic hydrocephalus, where he makes a drain from the spinal canal into the posterior part of the peritoneal cavity. This seems the most rational procedure which has yet been adopted, and some improvement has followed it occasionally. Washing out the spinal canal through canulas introduced into the subdural space in the brain and lumbar region for cases of cerebro-spinal meningitis and tetanus has been suggested by Murphy, of Chicago, and he brings forward good evidence in its favour.

*Typhoid Perforation* has, during the past year, been the subject of many communications. It is more than twenty years since Leyden first suggested, and Mickulicz carried out, an operation for this condition. Perforation of the intestine probably occurs once in every three deaths from enteric fever. Makins insists on the necessity for distinguishing between perforation with its own symptoms, and those of the subsequent and therefore too late peritonitis. The nurse's report of sudden change is very important, and of course the classical pain, tenderness, and local rigidity, loss of liver dulness (especially if the abdomen be not distended), and advance in the number of respirations. He makes the following statement, which in some institutions would not pass unchallenged: "For it frequently happens there is no alteration in the pulse until many hours have passed. No reliance should be placed upon blood examination." In his 32 cases the pulse is recorded as "no change" in 11. The blood-counts were taken in 18 cases, and were over 8,000 in only eight of these. Everyone now seems to agree in operation in most cases of perforation, and even in exploring in doubtful cases.

Great numbers of cases of *Prostatectomy* have been recorded during the year, and it has been noticed that the number of complications are many, such as hæmorrhage, sepsis, cystitis, pyelitis, uræmia, orchitis, persistent fistulæ, stricture, return of symptoms, and others. There has also been recorded a fair number of deaths; but in spite of all this the operation has proved itself one which has come to stay; but which particular operation is not yet decided. The general tendency in America, which is shared by the Mayo's and Bentley Squire, is to favour the perineal route, while Freyer and most English surgeons prefer the supra-pubic. Willy Meyer says: "Unassailable proof has been furnished to show that all three methods [the third being Bottini's operation] deserve to be recognised as standard procedures, each being capable of bringing permanent relief."

Young found that a certain percentage of his earlier cases turned out to be carcinomatous, and regretted not having done a more radical operation. He operates through the perinæum by an inverted V incision, exposing the urethra; a prostatic tractor is put into the bladder and opened out. The handle of this is depressed, and the membranous urethra divided; the anterior surface of the bladder is then cut across, exposing the trigone, and this is separated 1 c.m. in front of the urethral orifices and the whole mass removed. The bladder is then sewn to the urethra, the gap being entirely closed. With this operation he has had sufficient success to recommend it in early cases of carcinoma.

The journals have lately been full of papers on Graves's disease, and for the most part any surgical procedure is decried; but there can be no doubt there is a considerable amount of evidence in favour of partial removal of the thyroid in some cases. The Mayo's have operated on 40 cases, with six deaths. It must be remembered not only that some cases die from the disease itself, but also that other cases go on year after year as hopelessly chronic invalids. Hartley summarises the whole subject, and adding a number of statistics together, finds a mortality of 12.6 per cent. The good result in those cases operated on who do well is almost immediate, and is very marked.

*The Injection of Paraffin* has much improved in technique, chiefly by using wax of a lower melting-point, and consequently introducing it into the tissues in the semi-solid state; when this is done there is less liabilty of its becoming displaced, thus giving rise to unsightly lumps. Gersuny, of Vienna, who started this treatment, uses it also for flail joints, prolapse of the rectum, and some other deformities. He does not use general anæsthesia but injects cocaine previous to the injection of the wax. The writer of this article has seen a great number of cases operated on by Gersuny and noticed the absence of pain during them and the very good results obtained.

Professor Von Mosetig Moorhof, of Vienna, has for some years been using the method known as bone plugging. He makes free incisions, turning up a skin-muscle-periosteal flap, clearing out the disease with saws, spoons, burs, etc. When the cavities are quite dry and clean and all oozing has ceased, he pours in a warm mixture of iodoform, oil, and wax. After letting this set, he sews up by degrees, without drainage. As can be seen by skiagraphy, this material is gradually replaced by bone. Chronic osteomyelitis, tuberculous cavities, and those following on acute necrosis, are all dealt with in this way. Very good results have also been obtained in joint disease, which the professor says should be operated on early. Very free exposure is necessary, large flaps being better than vertical incisions, transverse section of the patella in the case of the knee-joint is advised, and in children the disease should be removed with a spoon and not with a saw, so as to avoid injury to the epiphysis. In the ankle the best exposure is from in front, cutting through all structures, including nerves and tendons—these are sewn together afterwards and with no bad results. Professor Moorhof has brought his bone surgery to a high pitch of excellence, and the writer can testify to the good results.

An interesting surgical point which has been brought out during the year is the advantage of sterilised clothes which the Japanese adopted on going into battle.

Much work which has been done on chest-surgery, especially in freeing the lung of its thick coat (peripneumotomy) in operations for chronic empyema.

In general technique surgeons are beginning to attach more importance to cleaning the skin at the time of operation, instead of previous preparation. J. B. Murphy recommends sitting up the patient at once after operation for peritonitis and claims he has by this means much diminished his mortality; and it is beginning to be recognised that silence on the part of the operators and onlookers is an important prophylaxis against sepsis.

